# Correction to “Body Dysmorphic Disorder Questionnaire: Arabic Adaptation and Validation in Dermatology Population”

**DOI:** 10.1002/mpr.70059

**Published:** 2026-01-23

Salameh, P., J. El Khoury, M. Hallal, et al. 2025. “Body Dysmorphic Disorder Questionnaire: Arabic Adaptation and Validation in Dermatology Population”, *International Journal of Methods in Psychiatric Research*, 34, no. 4: e70045. https://doi.org/10.1002/mpr.70045.

Table 1 and Table 2 were initially published with formatting errors. The published article has now been updated to display the correct formatting.

In paragraph 4 of the Discussion section, the citations of two references were incorrectly reversed. They have now been corrected and appear in the proper order in the published article.

We apologize for these errors.

